# Early vs. Late Onset Cardiac Injury and Mortality in Hospitalized COVID-19 Patients in Wuhan

**DOI:** 10.3389/fcvm.2021.645587

**Published:** 2021-05-28

**Authors:** Wei Sun, Yanting Zhang, Chun Wu, Shuyuan Wang, Yuji Xie, Danqing Zhang, Hongliang Yuan, Yongxing Zhang, Li Cui, Meng Li, Yiwei Zhang, Yuman Li, Jing Wang, Yali Yang, Qing Lv, Li Zhang, Philip Haines, Wen-Chih Wu, Mingxing Xie

**Affiliations:** ^1^Department of Ultrasound, Union Hospital, Tongji Medical College, Huazhong University of Science and Technology, Wuhan, China; ^2^Hubei Province Clinical Research Center for Medical Imaging, Wuhan, China; ^3^Hubei Province Key Laboratory of Molecular Imaging, Wuhan, China; ^4^Rhode Island Hospital, Warren Alpert Medical School of Brown University, Providence, RI, United States; ^5^Department of Medicine, Providence VA Medical Center, Brown University Warren Alpert Medical School, Providence, RI, United States

**Keywords:** COVID-19, cardiac injury, early, late, mortality

## Abstract

**Background:** Increasing evidence points to cardiac injury (CI) as a common coronavirus disease 2019 (COVID-19) related complication. The characteristics of early CI (occurred within 72 h of admission) and late CI (occurred after 72 h of admission) and its association with mortality in COVID-19 patients is unknown.

**Methods:** This retrospective study analyzed patients confirmed with COVID-19 in Union Hospital (Wuhan, China) from Jan 29th to Mar 15th, 2020. Clinical outcomes (discharge, or death) were monitored to April 15, 2020, the latest date of follow-up. Demographic, clinical, laboratory, as well as treatment and prognosis were collected and analyzed in patients with early, late CI and without CI.

**Results:** A total of 196 COVID-19 patients were included for analysis. The median age was 65 years [interquartile range (IQR) 56–73 years], and 112 (57.1%) were male. Of the 196 COVID-19 patients, 49 (25.0%) patients had early and 20 (10.2%) patients had late CI, 56.6% developed Acute-Respiratory-Distress-Syndrome (ARDS) and 43 (21.9%) patients died. Patients with any CI were more likely to have developed ARDS (87.0 vs. 40.2%) and had a higher in-hospital mortality than those without (52.2 vs. 5.5%, *P* < 0.001). Among CI subtypes, a significantly higher risk of in-hospital death was found in patients with early CI with recurrence [19/49 patients, adjusted odds ratio (OR) = 7.184, 95% CI 1.472–35.071] and patients with late CI (adjusted OR = 5.019, 95% CI 1.125–22.388) compared to patients with early CI but no recurrence.

**Conclusions:** CI can occur early on or late after, the initial 72 h of admission and is associated with ARDS and an increased risk of in-hospital mortality. Both late CI and recurrent CI after the initial episode were associated with worse outcomes than patients with early CI alone. This study highlights the importance of early examination and periodical monitoring of cardiac biomarkers, especially for patients with early CI or at risk of clinical deterioration.

## Introduction

Coronavirus disease 2019 (COVID-19) caused by the severe acute respiratory syndrome coronavirus 2 (SARS-CoV-2) has affected over 200 countries (1). With the increasing number of confirmed cases, the cardiovascular manifestations associated by this highly contagious viral infection have gained more and more attention. Several observational studies have found that between 7.2 and 37.5% of COVID-19 patients had cardiac injury (CI) which was associated with higher mortality in COVID-19 patients (2–11). However, a portion of CI does not occur on admission and the association of the timing of CI with prognosis is unknown. Furthermore, the clinical features and risk factors associated with early or late onset CI in COVID-19 patients have not been formally evaluated. The clinical sequence preceding and following CI at the time of admission may provide additional understanding of the pathogenesis associated with CI in COVID-19. Therefore, this study compared the clinical characteristics, risk factors and prognostic value of early vs. late onset of CI in COVID-19 patients.

## Methods

### Study Design and Participants

We performed this retrospective study at Union Hospital (Affiliated Tongji Medical College, Huazhong University of Science and Technology) Wuhan, China. The West Branch of Union Hospital was one of the major designated hospitals for critically ill COVID-19 patients. We enrolled 429 consecutive patients with confirmed COVID-19, according to the WHO interim guidance criteria (12), who were either discharged alive or died during hospitalization from Jan 29th to April 15th, 2020. Only participants who had high-sensitivity troponin I (hs-TNI) measured before and after 72 h from admission during their hospitalization were included in the study (233 patients excluded). The study was approved by the ethics committee of the Union hospital, Tongji Medical College, Huazhong University of Science and Technology. Per institutional policy, written informed consent was waived for all participants with emerging infectious diseases.

### Data Collection

Data were extracted from the electronic medical records including demographic information and clinical characteristics (i.e., vital signs, symptoms, laboratory findings, medical history, underlying comorbidities, treatments, complications, and outcomes) of the participants on admission and during hospitalization. The date of illness onset was defined as the day when symptoms of COVID-19 as defined by the World Health Organization (12) were appreciated. Laboratory measurements within and after 72 h of admission were collected. If multiple measurements were available, the patient's first abnormal measurements, both within and after 72 h, were recorded for the determination of the timing of the CI. The duration from the onset of admission to the onset of clinical complications and death in the hospital were also recorded. Clinical outcomes (discharge and mortality) were monitored up to April 15, 2020, the last date of follow-up. Complete hospitalization data was available in all patients included in the study.

### Timing of CI

The hs-TNI data for each patient were collected from admission to discharge or death. COVID-19 related CI was defined as the serum levels of cardiac high-sensitivity troponin I (hs-TNI) above the 99th percentile upper reference limit in a patient diagnosed with COVID-19 per Huang et al. and Shi et al. (2, 7–10). Early CI was defined as CI that occurred within 72 h of admission, whereas late CI was defined as occurring after 72 h of admission. We also defined a subgroup of recurrent CI within the early CI group as a second rise of hs-TNI value of >20% from its previous value after 72 h of admission.

### Non-cardiac Complications

Acute respiratory distress syndrome (ARDS) was defined according to the World Health Organization interim guidance criteria (13). Acute kidney injury was identified according to the KDIGO clinical practice guidelines as an increase in serum creatinine by ≥0.3 mg/dl (≥26.5 μmol/l) within 48 h or by 1.5 times of the baseline values (14). Coagulation dysfunction was defined as a >3-s prolongation of prothrombin time (PT) or a 5-s prolongation of activated partial thromboplastin time (APTT). Thrombocytopenia was characterized by a platelet count <125 × 10^9^/L (15).

### Statistical Analysis

Categorical variables were expressed as number (%), and continuous variables were expressed as mean ± standard deviation (SD) or median [interquartile range (IQR)]. The normality of the distribution was tested with the Shapiro-Wilk normality test. Differences among the three groups (without CI, early CI or late CI) were assessed by ANOVA for normally distributed and Kruskal–Wallis *H*-test for non-normally distributed continuous variables. Categorical variables were compared by Chi-square or Fisher exact test where applicable. In-hospital survival curves of four groups of patients with early but no recurrent CI, early with recurrent CI, late CI, and no CI were estimated with the Kaplan-Meier method and the groups compared with the log-rank test. Univariate and multivariate logistic regression analyses were used to determine the independent risk associated with each of the four groups (early but no recurrent CI, early with recurrent CI, late CI, and no CI) of patients with in-hospital death, adjusted by known risk factors of COVID-19 mortality in the literature [age, sex, respiratory rate, heart rate, SpO_2_, temperature, mean arterial pressure, coma, hypertension history, Lymphocyte count, C-reactive protein (CRP), and lactate dehydrogenase (LDH)] (16–18). All statistical analyses were performed with SPSS version 24.0 (Statistical Package for the Social Sciences, Chicago, Illinois), and STATA software version 10 (StataCorp, Texas, USA). A two-tailed *P*-value of <0.05 was considered statistically significant.

## Results

### Clinical Characteristics, Laboratory Findings, and Treatments Within 72 h of Admission

Among the 429 patients, 100 patients were excluded due to missing hs-TNI data during the entire hospitalization, 133 patients were excluded due to missing hs-TNI data within 72 h of admission, the remaining 196 patients were included for analysis. The median age was 65.0 years (IQR: 56.0–73.0 years), and 112 (57.1%) were men. Of 196 COVID-19 patients, 69/196 (35.2%) had evidence of CI during hospitalization: 49/196 (25.0%) patients had early CI and 20/196 (10.2%) patients had late CI. In addition, 19/49 (38.8%) patients with early CI had recurrent CI after 72 h of admission. Compared with patients without CI, patients with early and late CI were more often older, and had lower SpO_2_ on admission. They also had more comorbidities such as hypertension and underlying cardiac disease.

Compared with non-CI group, patients with early and late CI presented with more abnormal laboratory findings within 72 h of admission including lower lymphocyte and platelet counts, higher inflammation-related indices [CRP, procalcitonin (PCT)] and further elevations in liver and renal function indices.

Concerning the treatment of the 196 patients within 72 h of admission, there was no difference in the antiviral (*P* = 0.551) or antibiotic therapy (*P* = 0.235) among these three groups. However, compared with the non-CI group, more patients with early and late CI received glucocorticoid therapy (18.9 vs. 38.8% and 30.0%, *P* = 0.021), high-flow oxygen (18.1 vs. 69.4% and 80.0%, *P* < 0.001), invasive mechanical ventilation (0.8 vs. 4.1% and 15.0%; *P* = 0.005), non-invasive mechanical ventilation (0.0 vs. 8.2% and 15.0%; *P* < 0.001), and more subjects were transferred to the intensive care unit (ICU) (0.8 vs. 10.2% and 5.0%; *P* = 0.007) (Table 1).

**Table 1:** Comparisons of demographics, clinical characteristics and laboratory examinations on admission within 72 h among the three groups.

	**Total population (*n* = 196)**	**Without CI(*n* = 127)**	**Early CI (*n* = 49)**	**Late CI(*n* = 20)**	***P*-value**
Age (years)	65.0 (56.0, 73.0)	61.0 (49.0, 69.0)	71.0 (66.0, 77.0)*	69.0 (59.3, 72.8)	<0.001
Gender					0.458
Male, *n* (%)	112 (57.1)	70 (51.1)	28 (57.1)	14 (70.0)	
Female, *n* (%)	84 (42.9)	57 (44.9)	21 (42.9)	6 (30.0)	
Smoking, *n* (%)	22 (11.2)	16 (12.6)	5 (10.2)	1 (5.0)	0.537
**Vital signs**					
Temperature (°C)	38.0 (36.7, 38.7)	38.0 (36.7, 38.7)	37.9 (36.7, 38.5)	38.9 (38.1, 39.5)*^#^	0.004
Respiratory rate (breaths/min)	20.0 (20.0, 25.0)	20.0 (20.0, 24.0)	23.0 (20.0, 30.0)*	22.0 (20.0, 25.0)	0.008
Heart rate (bpm)	89.0 (80.0, 101.0)	87.0 (80.0, 98.0)	95.0 (81.0, 110.5)	92.0 (83.0, 106.5)	0.068
SBP (mmHg)	133.6 ± 19.9	132.7 ± 18.8	135.3 ± 22.6	135.5 ± 20.7	0.547
DBP (mmHg)	80.4 ± 13.1	81.3 ± 12.0	78.7 ± 15.5	79.0 ± 18.8	0.182
Mean arterial pressure (mmHg)	98.2 ± 13.9	98.4 ± 12.9	97.6 ± 16.2	98.6 ± 15.0	0.806
SpO_2_ (%)	97.0 (94.0, 99.0)	98.0 (95.0, 99.0)	95.0 (89.5, 98.0)*	94.0 (87.5, 98.0)*	<0.001
**Common initial symptoms**					
Fever, *n* (%)	151 (77.0)	101 (79.5)	33 (67.3)	17 (85.0)	0.162
Cough, *n* (%)	113 (57.7)	78 (61.4)	23 (46.9)	12 (60.0)	0.214
Fatigue, *n* (%)	89 (45.4)	52 (40.9)	28 (57.1)	9 (45.0)	0.154
Dyspnea, *n* (%)	87 (44.4)	50 (39.4)	29 (59.2)	8 (40.0)	0.055
Chest tightness/chest pain, *n* (%)	75 (38.3)	50 (39.4)	17 (34.7)	8 (40.0)	0.837
Diarrhea, *n* (%)	21 (10.7)	17 (13.4)	4 (8.2)	0 (0.0)	0.056
Headache, *n* (%)	11 (5.6)	8 (6.3)	2 (4.1)	1 (5.0)	0.893
Coma, *n* (%)	9 (4.6)	2 (1.6)	6 (12.2)*	1 (5.0)	0.011
**Comorbidities**					
Hypertension, *n* (%)	87 (44.4)	48 (37.8)	30 (61.2)*	9 (45.0)	0.020
Diabetes mellitus, *n* (%)	29 (14.8)	14 (11.0)	10 (20.4)	5 (25.0)	0.130
Cardiac disease, *n* (%)	32 (16.3)	12 (9.4)	16 (32.7)*	4 (20.0)	0.001
Cerebral infarction, *n* (%)	15 (7.7)	7 (5.5)	6 (12.2)	2 (10.0)	0.234
Malignancy, *n* (%)	9 (4.6)	5 (3.9)	2 (4.1)	2 (10.0)	0.417
Chronic liver disease, *n* (%)	4 (2.0)	1 (0.8)	1 (2.0)	2 (10.0)*	0.037
Chronic kidney disease, *n* (%)	6 (3.1)	2 (1.6)	4 (8.2)	0 (0.0)	0.056
Chronic obstructive pulmonary disease, *n* (%)	8 (4.1)	3 (2.4)	4 (8.2)	1 (5.0)	0.173
Symptom onset to hospital admission (days)	14.0 (8.0, 20.0)	15.0 (10.0, 21.0)	10.0 (5.0, 14.5)*	10.0 (7.0, 15.0)*	<0.001
**Laboratory findings on admission (within 72 h)**					
White blood cells ( ×10^9^/L)	7.0 (5.5, 9.2) 195/196	6.4 (5.2, 7.9) 126/127	8.9 (6.8, 10.9)* 49/49	7.0 (5.2, 9.6) 20/20	<0.001
Lymphocyte (%)	15.9 (7.4, 25.9) 195/196	22.1 (13.8, 29.3) 126/127	6.6 (4.6, 15.9)* 49/49	10.4 (6.0, 17.4)* 20/20	<0.001
Neutrophil ( ×10^9^/L)	5.3 (3.7, 7.4) 195/196	4.3 (3.1, 6.0) 126/127	7.2 (5.9, 10.1)* 49/49	5.8 (4.5, 7.7) 20/20	<0.001
Platelets ( ×10^9^/L)	205.0 (152.0, 262.0) 195/196	214.0 (174.5, 273.5) 126/127	179.0 (117.5, 262.5)* 49/49	167.5 (108.5, 245.0) 20/20	0.008
Hemoglobin (g/L)	124.0 (112.0, 135.0) 195/196	125.0 (110.8, 135.3) 126/127	121.0 (101.0, 135.0) 49/49	128.5 (116.3, 133.3) 20/20	0.452
CRP (mg/L)	32.0 (3.4, 75.9) 195/196	6.5 (1.9, 55.2) 126/127	62.4 (36.3, 109.9)* 49/49	71.0 (33.8, 114.6)* 20/20	<0.001
PCT (ng/ml)	0.11 (0.05, 0.24) 176/196	0.06 (0.04, 0.12) 114/127	0.40 (0.14, 0.60)* 42/49	0.15 (0.11, 0.28)* 20/20	<0.001
**Coagulation function index**					
D-dimer (μg/mL)	1.11 (0.35, 4.27) 186/196	0.75 (0.29, 2.39) 120/127	2.73 (1.28, 8.00)* 46/49	1.05 (0.46, 6.35) 20/20	<0.001
PT (s)	13.4 (12.6, 14.3) 186/196	13.1 (12.4, 14.0) 120/127	14.2 (13.2, 15.1)* 46/49	13.5 (13.0, 14.2) 20/20	<0.001
APTT (s)	37.4 (33.1, 42.2) 186/196	36.0 (32.5, 39.9) 120/127	38.7 (33.9, 44.9) 46/49	38.1 (32.4, 44.1) 20/20	0.274
**Liver function index**					
Total protein (g/L)	64.1 (58.9, 67.6) 196/196	64.4 (58.8, 67.7) 127/127	60.7 (56.2, 64.5)* 49/49	62.1 (60.0, 66.8) 20/20	0.002
Albumin (g/L)	31.7 (26.8, 37.4) 196/196	32.1 (27.3, 37.7) 127/127	28.2 (24.4, 32.1)* 49/49	27.4 (25.3, 31.8)* 20/20	<0.001
AST (U/L)	31.5 (23.0, 48.0) 196/196	28.0 (20.0, 43.0) 127/127	39.0 (29.0, 59.5)* 49/49	47.0 (33.8, 76.8)* 20/20	<0.001
ALT (U/L)	36.0 (23.0, 54.8) 196/196	32.5 (21.0, 50.0) 127/127	38.0 (23.0, 56.5) 49/49	38.5 (30.3, 78.8) 20/20	0.262
Total bilirubin (μmol/L)	11.2 (8.7, 16.9) 196/196	10.9 (8.3, 15.2) 127/127	14.2 (9.9, 21.3) 49/49	10.5 (7.8, 18.7) 20/20	0.110
Direct bilirubin (μmol/L)	3.8 (2.7, 5.6) 196/196	3.4 (2.5, 5.1) 127/127	4.8 (3.2, 7.6)* 49/49	4.3 (2.7, 5.9) 20/20	0.003
LDH (U/L)	250.5 (182.3, 403.0) 196/196	227.0 (174.3, 352.5) 127/127	388.0 (250.0, 597.0)* 49/49	492.0 (320.0, 665.3)* 20/20	<0.001
**Kidney function index**					
BUN (mmol/L)	5.2 (4.1, 7.3) 196/196	4.8 (3.7, 6.3) 127/127	7.0 (5.0, 11.2)* 49/49	5.7 (4.1, 7.5) 20/20	<0.001
Serum creatinine (μmol/L)	68.4 (56.4, 82.5) 196/196	64.3 (54.4, 75.2) 127/127	75.3 (61.4, 98.1)* 49/49	68.6 (62.5, 82.2) 20/20	0.008
K+ (mmol/L)	4.0 (3.5, 4.3) 195/196	3.9 (3.5, 4.2) 126/127	4.0 (3.5, 4.4) 49/49	4.0 (3.5, 4.6) 20/20	0.427
Na+ (mmol/L)	138.9 (136.9, 141.1) 195/196	139.0 (137.3, 141.2) 126/127	139.9 (136.1, 144.0) 49/49	138.8 (135.8, 140.5) 20/20	0.558
**Cardiac injury index**					
hs-TNI (ng/L)	8.3 (2.7, 27.0) 196/196	3.9 (2.0, 9.8) 127/127	86.4 (44.9, 378.8)* 49/49	10.8 (7.5, 16.2)*^#^ 20/58	<0.001
CK-MB (U/L)	12.0 (10.0, 17.0) 170/196	11.0 (9.0, 15.0) 108/127	16.0 (10.0, 26.3)* 42/49	16.0 (10.3, 21.8)* 20/20	<0.001
BNP (pg/ml)	40.7 (15.0, 128.0) 152/196	30.1 (10.5, 84.1) 91/127	153.4 (46.0, 431.6)* 44/49	40.6 (22.9, 122.4)^#^ 17/20	<0.001
**Treatments on admission (within 72 h)**					
Antiviral therapy, *n* (%)	147 (75.0)	94 (74.0)	36 (73.5)	17 (85.0)	0.551
Antibiotic therapy, *n* (%)	107 (54.6)	61 (48.0)	31 (63.3)	15 (75.0)	0.235
Glucocorticoid therapy, *n* (%)	49 (25.0)	24 (18.9)	19 (38.8)*	6 (30.0)	0.021
Immunoglobulin, *n* (%)	21 (10.7)	10 (7.9)	6 (12.2)	5 (25.0)	0.104
ACEI/ARB, *n* (%)	8 (4.1)	4 (3.1)	4 (8.2)	0 (0.0)	0.258
Oxygen therapy, *n* (%)	132 (67.3)	73 (57.5)	42 (85.7)*	17 (85.0)	<0.001
High-flow oxygen, *n* (%)	73 (37.2)	23 (18.1)	34 (69.4)*	16 (80.0)*	<0.001
IMV, *n* (%)	6 (3.1)	1 (0.8)	2 (4.1)	3 (15.0)*	0.005
NIMV, *n* (%)	7 (3.6)	0 (0.0)	4 (8.2)*	3 (15.0)*	<0.001
ICU transfer, *n* (%)	7 (3.6)	1 (0.8)	5 (10.2)*	1 (5.0)	0.007

* *P < 0.05, vs. without CI*;

# *P < 0.05, vs. early CI; ACE-I, angiotensin-converting enzyme inhibitors; ALT, alanine aminotransferase; APTT, activated partial thromboplastin time; ARB, angiotensin II receptor blockers; AST, aspartate aminotransferase; BNP, B-type natriuretic peptide; BUN, blood urea nitrogen; CI, cardiac injury; CK-MB, creatine kinase muscle-brain; CRP, C-reactive protein; DBP, diastolic blood pressure; hs-TNI, hypersensitive troponin I; ICU, intensive care unit; IMV, invasive mechanical ventilation; IQR, interquartile range; LDH, lactate dehydrogenase; NIMV, non-invasive mechanical ventilation; PCT, procalcitonin; PT, prothrombin time; SBP, systolic blood pressure; SD, standard deviation*.

### Timing of CI and Non-cardiac Complications After 72 h of Admission

Major complications after 72 h included ARDS [111/196 (56.6%)], coagulation dysfunction [57/193 (29.5%)], late CI [20/196 (10.2%)] and acute kidney injury [33/193 (17.1%)]. Patients with early and late CI were more likely to have developed ARDS, ICU transfer and receive invasive mechanical ventilation (IMV) during their hospitalization compared to the non-CI group (Table 2). A majority, 87.0% (60/69) of patients with CI vs. 40.2% (51/127) without CI, developed ARDS. Overall, the median time from admission to ARDS was 4 days, to acute kidney injury was 7 days, to late CI was 11 days, and to coagulation dysfunction was 11 days for all patients.

**Table 2:** Comparisons of additional treatment, complications, and prognosis after 72h of admission among the three groups.

**Variables**	**Total population**	**Without CI**	**Early CI**	**Late CI**	***P*-value**
	**(*n* = 196)**	**(*n* = 127)**	**(*n* = 49)**	**(*n* = 20)**	
**Additional treatment after admission**					
Antiviral therapy, *n* (%)	36 (18.4)	24 (18.9)	9 (18.4)	3 (15.0)	1.000
Antibiotic therapy, *n* (%)	50 (25.5)	31 (24.4)	14 (28.6)	5 (25.0)	0.882
Glucocorticoid therapy, *n* (%)	48 (24.5)	26 (20.5)	11 (22.4)	11 (55.0)*^#^	0.007
Immunoglobulin, *n* (%)	49 (25.0)	22 (17.3)	14 (28.6)	13 (65.0)*^#^	<0.001
ACEI/ARB, *n* (%)	17 (8.7)	12 (9.4)	3 (6.1)	2 (10.0)	0.733
Oxygen therapy, *n* (%)	34 (17.3)	26 (20.5)	5 (10.2)	3 (15.0)	0.273
High-flow oxygen, *n* (%)	51 (26.0)	42 (33.1)	5 (10.2)*	4 (20.0)	0.006
IMV, *n* (%)	33 (16.8)	8 (6.3)	13 (26.5)*	12 (60.0)*^#^	<0.001
NIMV, *n* (%)	16 (8.2)	8 (6.3)	5 (10.2)	3 (15.0)	0.234
ICU transfer, *n* (%)	25 (12.8)	6 (4.7)	7 (14.3)	12 (60.0)*^#^	<0.001
**Complications**					
Cardiac injury (CI)					
Early CI, *n* (%)	49/196 (25.0)	/	49/49(100)	0/20 (0)	/
Recurrent CI, *n* (%)	19/49 (38.8)	/	19/49 (38.8)	/	/
Late CI, *n* (%)	20/196 (10.2)	/	/	20/20 (100)	/
ARDS, *n* (%)	111/196 (56.6)	51/127 (40.2)	40/49 (81.6)*	20/20 (100)*	<0.001
Coagulation dysfunction, *n* (%)	57/193 (29.5)	21/124 (16.9)	22/49 (44.9)*	14/20 (70.0)*	<0.001
Acute kidney injury, *n* (%)	33/193 (17.1)	8/125 (6.4)	17/48 (35.4)*	8/20 (40.0)*	<0.001
**Time from admission to complications onset**					
Cardiac injury (CI)					
Early CI (days)	1 (0, 1)	**/**	1 (0, 1)	/	/
Recurrent CI (days)	7 (5, 16)	/	7 (5, 16)	/	/
Late CI (days)	11 (5, 22)	/	/	11 (5, 22)	/
ARDS (days)	4 (2, 9)	6 (3, 12)	2 (1, 8)*	7 (2, 8)	0.014
Coagulation dysfunction (days)	11 (1, 20)	11 (1, 19)	8 (1, 15)	23 (8, 25)^#^	0.02
Acute kidney injury (days)	7 (3, 14)	10 (3, 17)	4 (2, 7)	12 (6, 21)	0.065
**Prognosis**					
Death, *n* (%)	43 (21.9)	7 (5.5)	23 (46.9)*	13 (65.0)*	<0.001

*Data are median (IQR) or n (%)*.

* *P < 0.05, vs. without CI*;

# *P < 0.05, vs. early CI; ACE-I, angiotensin-converting enzyme inhibitors; ARB, angiotensin II receptor blockers; ARDS, acute respiratory distress syndrome; ICU, intensive care unit; IMV, invasive mechanical ventilation; IQR, interquartile range; NIMV, non-invasive mechanical ventilation*.

**Figure 1:**
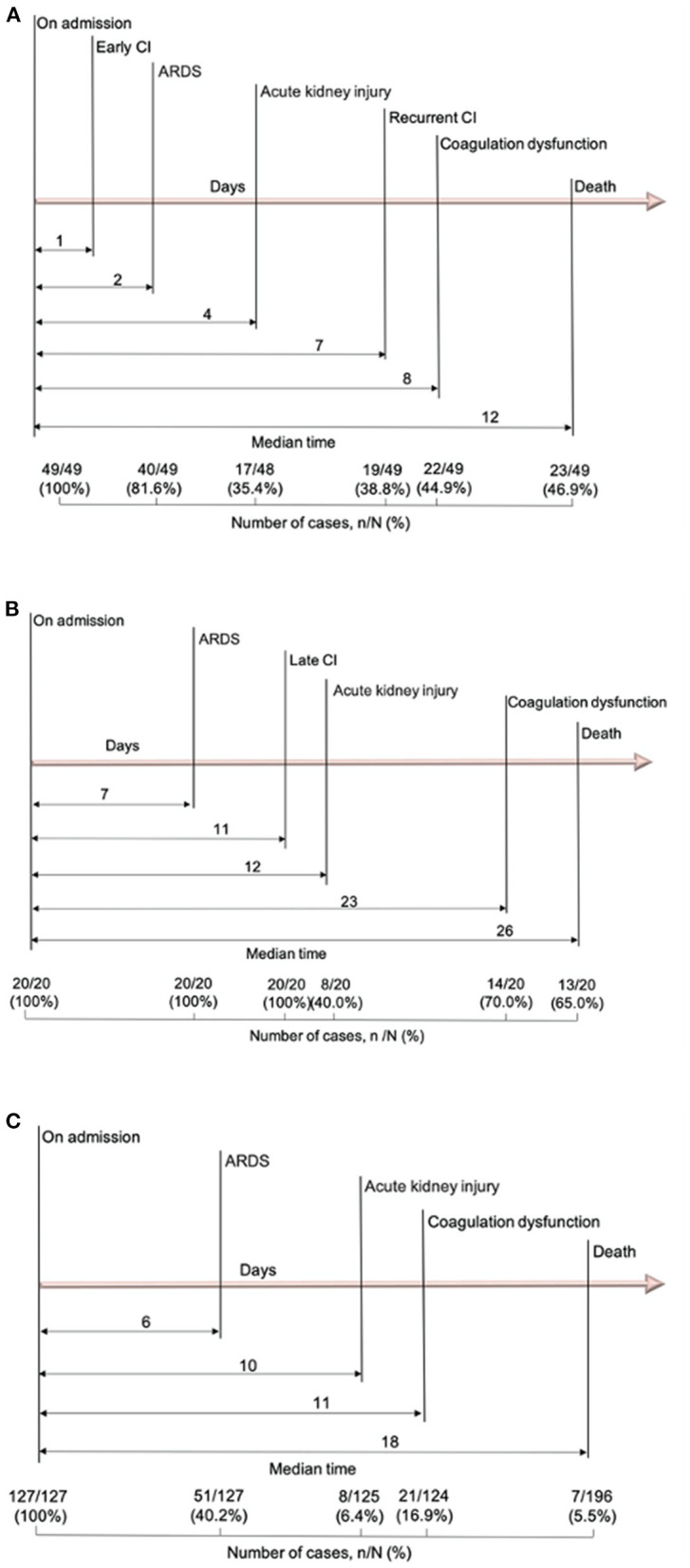
Timeline of COVID-19 patients after admission. **(A)** Timeline of COVID-19 patients with early CI; **(B)** Timeline of COVID-19 patients with late CI; **(C)** Timeline of COVID-19 patients without CI. COVID-19, coronavirus disease 2019; CI, cardiac injury.

For the early CI group (*n* = 49), the median time from admission to CI was 1 day (IQR 0–1 day), to the onset of ARDS (81.6%) was 2 days (IQR 1–8 days), and to the onset of recurrent CI [19/49 (38.8%)] was 7 days (IQR 5–16 days) for affected patients (Figure 1A). For the late CI group [20/196 (10.2%)], the median time from admission to the onset of ARDS (100%) was 7 days (IQR 2–8 days) and the onset of late CI was 11 days (IQR 5–22 days) (Figure 1B). Conversely, for the non-CI group [127/196 (64.8%)], the median time from admission to the onset of ARDS (40.2%) was 6 days (IQR 3–12 days) (Figure 1C).

**Figure 2:**
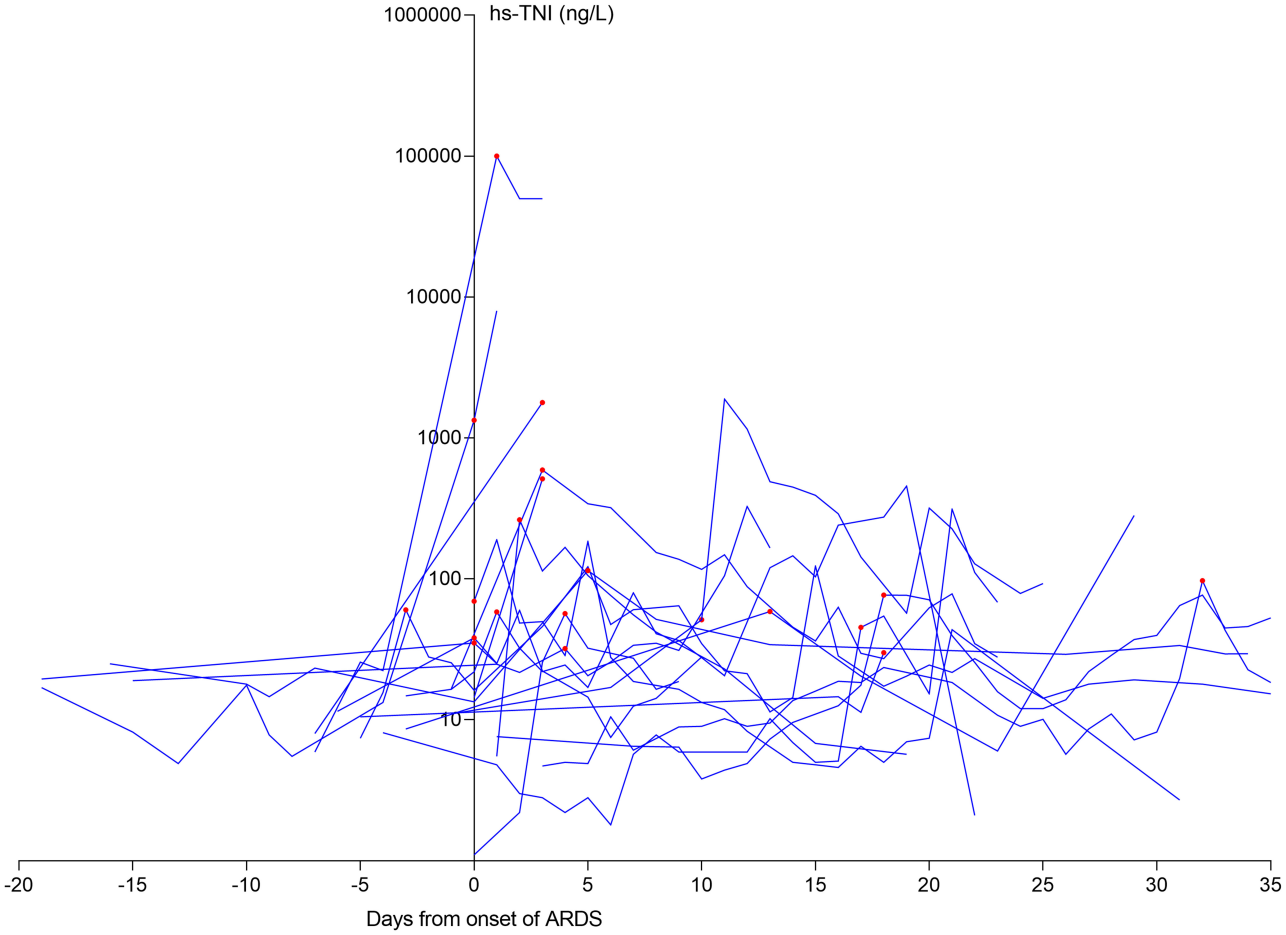
The dynamic profile of hs-TNI levels in late CI patients in relation to ARDS onset. 15/20 (75.0%) patients suffering from late CI after the onset of ARDS. The levels of hs-TN were log transformed. ARDS, acute respiratory distress syndrome; CI, cardiac injury; hs-TNI, high-sensitivity troponin I.

Using the onset of ARDS as the temporal reference point, 15/20 (75%) patients with late CI had ARDS before their CI (Figure 2), with a median time between ARDS and CI of 4 days (IQR 3–17 days).

### Cardiac Injury and Mortality

**Figure 3:**
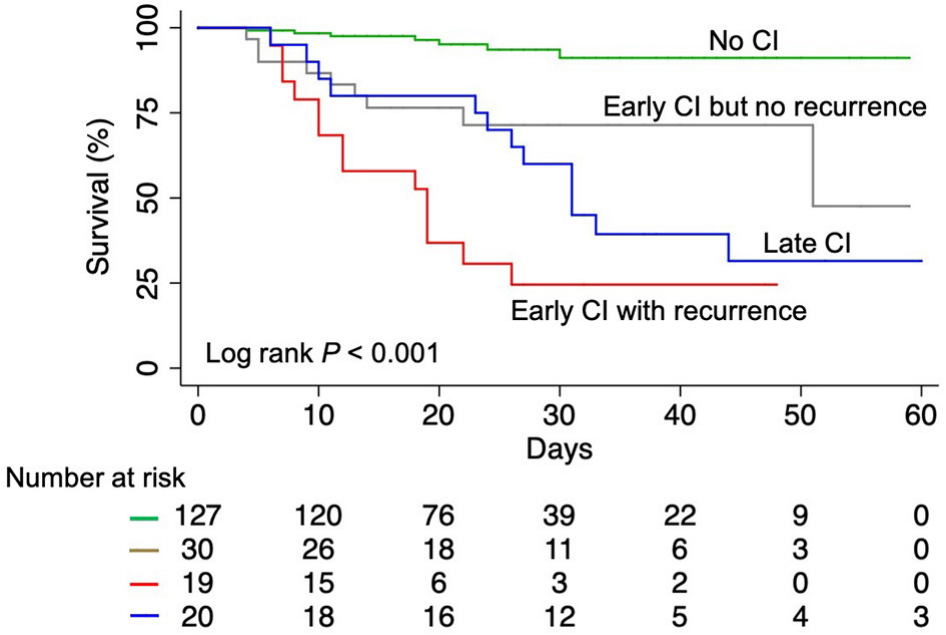
Kaplan-Meier survival curves for COVID-19 patients. COVID-19, coronavirus disease 2019; CI, cardiac injury.

As of April 15, 2020, 153 patients (78.1%) were discharged and 43 patients (21.9%) died in the hospital. Patients with any CI had significantly higher in-hospital mortality than those without (52.2 vs. 5.5%, *P* < 0.001) (Figure 3). Among CI subtypes, multivariable regression modeling showed that compared to patients with early CI but no recurrence, a significantly higher risk of in-hospital death was found in patients with early CI with recurrence [odds ratio (OR) = 7.184, *P* = 0.015] and patients with late CI (OR = 5.019, *P* = 0.034) (Table 3).

**Table 3:** Univariate and multivariate logistic regression analysis of factors associated with in-hospital mortality of COVID-19 patients.

	**Univariate**		**Multivariate**	
**Factors**	**Unadjusted OR**	***P*-value**	**Adjusted OR**	***P*-value**
	**(95% confidence interval)**		**(95% confidence interval)**	
Age group (years)				
<45	1 (ref)			
45–54	0.680 (0.040, 11.632)	0.79		
55–64	4.675 (0.559, 39.116)	0.155		
65–74	7.650 (0.944, 61.980)	0.057		
>74	6.581 (0.787, 55.045)	0.082		
Sex				
Female	1 (ref)		1 (ref)	
Male	3.632 (1.632, 8.086)	0.002	8.828 (2.463, 31.643)	0.001
Respiratory rate (breaths/min)				
12–24	1 (ref)		1 (ref)	
>24	3.667 (1.804, 7.451)	<0.001	3.773 (1.188, 11.983)	0.024
Heart rate (bpm)				
70–109	1 (ref)			
40–69	2.321 (0.547, 9.845)	0.253		
110–139	3.095 (1.261, 7.600)	0.014		
140–179	4.643 (0.627, 34.377)	0.133		
SpO_2_ (%)				
>89	1 (ref)		1 (ref)	
<75	24.833 (2.661, 231.712)	0.005	8.264 (0.694, 98.332)	0.095
75–85	31.042 (6.405, 150.435)	<0.001	11.129 (1.341, 92.388)	0.026
86–89	31.042 (3.474, 277.332)	0.002	74.421(3.121, 1774.444)	0.008
Temperature (°C)				
<37.2	1 (ref)			
37.2–38.9	1.991 (0.855, 4.633)	0.11		
>38.9	2.031 (0.741, 5.564)	0.168		
Mean arterial pressure (mmHg)				
70–109	1 (ref)			
50–69	8.065 (0.708, 91.829)	0.094		
110–129	1.578 (0.664, 3.748)	0.302		
130–159	1.008 (0.109, 9.340)	0.994		
Coma (yes vs. no) (9 vs. 320)	8.108 (1.937, 33.944)	0.004		
Hypertension (yes vs. no) (139 vs. 190)	2.974 (1.467, 6.029)	0.003		
Lymphocytes (%)				
≥20	1 (ref)			
<20	10.551 (3.594, 30.980)	<0.001		
CRP (mg/L)				
≤ 8	1 (ref)			
>8	17.500 (4.086, 74.956)	<0.001		
LDH (U/L)				
≤ 245	1 (ref)			
>245	8.129 (3.239, 20.398)	<0.001		
Type of CI				
Early CI but no recurrence	1 (ref)		1 (ref)	
Early CI with recurrence	6.533 (1.807, 23.627)	0.004	7.184 (1.472, 35.071)	0.015
Late CI	4.333 (1.298, 14.471)	0.017	5.019 (1.125, 22.388)	0.034
No CI	0.136 (0.046, 0.405)	<0.001	0.119 (0.030, 0.475)	0.003

*OR, odds ratio; CI, cardiac injury; COVID-19, coronavirus disease 2019; CRP, C-reactive protein; LDH, lactate dehydrogenase*.

## Discussion

In this cohort of 196 hospitalized COVID-19 patients from Wuhan, China, we found 35.2% had evidence of CI during hospitalization that included 25.0% of patients with early CI and 10.2% patients with late CI. In addition, there were 38.8% of patients with early CI who also had recurrent CI. Patients with any CI had significantly higher incidence of ARDS and in-hospital mortality than those without. Moreover, among CI subtypes, a significantly higher risk of in-hospital death was found in patients with early CI with recurrence and patients with late CI compared to patients with early CI and no recurrence.

Increasingly, researchers are reporting CI in COVID-19 patients, with the prevalence varying from 7.2 to 37.5% (4, 7, 8, 11). In our study, a remarkable 35.2% of patients had any CI. The pathogenesis of CI associated with COVID-19 is still unknown partly due to a dearth of autopsy or biopsy reported in these patients. The following potential mechanisms for CI have been proposed. The first possibility is direct myocardial damage by the virus, because angiotensin-converting enzyme 2 has been identified as a functional receptor for coronaviruses, which is also expressed abundantly in the myocardium (19, 20). Mixed literature from autopsy and biopsy case series were reported without conclusive evidence yet (21–25). The second mechanism is presumably the systemic inflammatory cytokine response, namely cytokine storm (26, 27). It can cause the proliferation of highly pro-inflammatory CCR4+CCR6+Th17 cells amongst the CD4+T cells, the expression of high concentrations of cytotoxic particles in CD8+T cells and over activation of T cells in general, all of which lead to a stronger inflammatory response in return (3, 21). Inflammation can in turn lead to thromboembolic complications (28). Our study showed that inflammation-related indices (CRP, PCT) were higher in the CI group compared to the non-CI group. Another mechanism is CI related to hypoxia. The balance between the oxygen demand and supply of the myocardium is disrupted during acute hypoxia. A cascade of cellular, biochemical and inflammatory reactions can occur during hypoxia, eventually causing myocardial apoptosis (29). Acute severe hypoxia can also trigger a systemic inflammatory response (30). In this study, patients with early or late CI had lower level of SpO_2_ on admission and higher incidence of ARDS compared to the non-CI group. There was a large timing overlap between CI and ARDS in the early CI group and 75% of patients with late CI were preceded by ARDS which supports a strong relationship between CI and hypoxia. Lastly, antiviral drugs can cause cardiac insufficiency, arrhythmia or other cardiovascular disorders with variable individual susceptibility (4, 31). In the present study, almost all patients (93.4% of COVID-19 patients) were administrated with antiviral drugs either on admission or after admission for which the opportunity of individual susceptibility and/or interaction with the underlying comorbid conditions could contribute to onset of late CI or recurrence of the initial one. In addition, patients in the CI group were more likely to have received glucocorticoid therapy. The relationship between glucocorticoid therapy and cardiac injury remains controversial and is under investigation (32). On the other hand, it is also likely that patients with any CI had higher disease severity for which more treatment was administered. This may suggest that the CI after admission was a sign of disease severity and/or progression.

Recent studies have demonstrated that CI was associated with increased mortality in COVID-19 patients (7, 8, 33). However, to the best of our knowledge, this is the first study to depict the clinical characteristics of both early CI and late CI and their association with in-hospital mortality. Our study showed that early CI was an independent predictor of death in COVID-19 patients even after accounting for variables that have proven prognostic value in the risk stratification of acutely ill patients, such as respiratory rate, heart rate, SpO_2_, temperature, mean arterial pressure, and coma (16, 34). Cardiac injury remained significant after accounting for potential confounding laboratory variables with proven prognostic value in COVID hospitalizations such as lymphocyte count, LDH, CRP (17). This is important because clinicians should not consider patients being out of danger despite absence of CI in the first 72 h since it can occur late. Similarly, a fall after the early rise in Trop I should not translate into a lower risk status since recurrence of CI can occur. Conversely, we also found that CI is closely related to the occurrence of ARDS as opposed to a primary cardiac event. Accordingly, our study suggested the need for a more systematic assessment of cardiac troponins for risk stratification of COVID-19 patients on admission, with repeat measures for those who already have initial CI or who are at risk for clinical deterioration. With the advent of multiple therapies now to reduce the morbidity and mortality of COVID-19 (35–37), early identification of higher risk patients with cardiac biomarkers may be helpful to balance the cost vs. the effect of therapy.

## Limitations

Our study has some limitations. First, this was a relatively small sample size and single-center retrospective observational study for which residual confounding cannot be excluded and we did not include all the potential factors associated with mortality in our study. Second, only 196 patients both had the data of hs-TNI within and after 72 h of admission, which may have underestimated the true incidence of CI in our study. Therefore, future studies should be multi-centered, with larger sample size and systematic data collection, in order to promote a more comprehensive understanding of the association between cardiac injury and mortality in hospitalized patients with COVID-19.

## Conclusions

CI is a common condition that can occur early on or late after, the initial 72 h of admission and is associated with ARDS and an increased risk of in-hospital mortality. Both late CI and recurrent CI after the initial episode were associated with worse outcomes than patients with early CI alone. This study highlights the importance of early examination and periodical monitoring of cardiac biomarkers to identify predictors and markers of clinical deterioration in COVID-19 patients to guide intervention.

## Data Availability Statement

The original contributions presented in the study are included in the article/supplementary material, further inquiries can be directed to the corresponding authors.

## Ethics Statement

The studies involving human participants were reviewed and approved by the Ethics Committee of Tongji Medical College, Huazhong University of Science and Technology. Written informed consent was waived for all participants with emerging infectious diseases. Written informed consent for participation was not required for this study in accordance with the national legislation and the institutional requirements.

## Author Contributions

MX, W-CW, PH, and LZ: conception and design of study. WS, YaZ, CW, SW, DZ, HY, YoZ, LC, YL, JW, YY, and QL: acquisition of data. YaZ, WS, SW, ML, and YiZ: analysis and/or interpretation of data. LZ, WS, YaZ, and CW: drafting the manuscript. MX, W-CW, PH, and LZ: revising the manuscript critically for important intellectual content. All authors contributed to the article and approved the submitted version.

## Conflict of Interest

The authors declare that the research was conducted in the absence of any commercial or financial relationships that could be construed as a potential conflict of interest.
